# Construction of sized eukaryotic cDNA libraries using low input of total environmental metatranscriptomic RNA

**DOI:** 10.1186/1472-6750-14-80

**Published:** 2014-09-03

**Authors:** Rajiv Kumar Yadav, Florian Barbi, Antoine Ziller, Patricia Luis, Roland Marmeisse, M Sudhakara Reddy, Laurence Fraissinet-Tachet

**Affiliations:** 1 Ecologie Microbienne, UMR CNRS 5557, USC INRA 1364, Université Lyon 1, Université de Lyon, Villeurbanne, France; 2 Department of Biotechnology, Thapar University, Patiala 147004, Punjab, India

**Keywords:** Metatranscriptomics, cDNA, cDNA library, mRNA, Gel electrophoresis

## Abstract

**Background:**

Construction of high quality cDNA libraries from the usually low amounts of eukaryotic mRNA extracted from environmental samples is essential in functional metatranscriptomics for the selection of functional, full-length genes encoding proteins of interest. Many of the inserts in libraries constructed by standard methods are represented by truncated cDNAs due to premature stoppage of reverse transcriptase activity and preferential cloning of short cDNAs.

**Results:**

We report here a simple and cost effective technique for preparation of sized eukaryotic cDNA libraries from as low as three microgram of total soil RNA dominated by ribosomal and bacterial RNA. cDNAs synthesized by a template switching approach were size-fractionated by two dimensional agarose gel electrophoresis prior to PCR amplification and cloning. Effective size selection was demonstrated by PCR amplification of conserved gene families specific of each size class. Libraries of more than one million independent inserts whose sizes ranged between one and four kb were thus produced. Up to 80% of the insert sequences were homologous to eukaryotic gene sequences present in public databases.

**Conclusions:**

A simple and cost effective technique has been developed to construct sized eukaryotic cDNA libraries from environmental samples. This technique will facilitate expression cloning of environmental eukaryotic genes and contribute to a better understanding of basic biological and/or ecological processes carried out by eukaryotic microbial communities.

## Background

The numerous eukaryotic microorganisms present in the environment potentially represent a rich source of genes encoding for novel enzymes or other proteins of interest in biotechnology. In this respect, functional metatranscriptomics has been demonstrated as a powerful tool in discovery of these genes [1-6]. Functional metatranscriptomics first requires the extraction of total RNA from environmental samples. Eukaryotic 3′ polyadenylated (poly-A) messenger RNAs can then be purified from total RNA to remove the ribosomal RNA, other non-coding RNAs as well as the bacterial mRNAs that largely dominate environmental metatranscriptomes [1,4,5]. Poly-A mRNAs are then converted into cDNAs which are cloned in an appropriate expression vector. Such eukaryotic-specific environmental cDNA libraries, first described by Grant *et al*. [7], thus encompass protein coding genes expressed by the different eukaryotic microorganisms present in the original environmental sample [1,7]. Genes of interest can then be screened by expressing them in an appropriate eukaryotic system such as the yeast *Saccharomyces cerevisiae*[1,3-5].

Expression cloning of eukaryotic genes using reverse transcribed poly-A mRNA is a fundamental technology in molecular biology. However, obtaining libraries enriched in long cDNAs remains challenging for the production of functional proteins. The first step is the reverse transcription of mRNAs into cDNAs. This step is adversely affected by many factors and as a consequence, a large proportion of long mRNAs (e.g. larger than 1 kb) is represented by 5′ truncated cDNAs [8]. Being small in size, these truncated cDNAs are preferentially amplified and cloned. Furthermore, functional metatranscriptomics most often involves the use of very low quantities of environmental eukaryotic mRNAs. This necessitates a highly efficient cDNA cloning approach which can make long eukaryotic transcripts available for functional studies.

Various library construction methods that enrich long cDNAs have been proposed. For example, several approaches that use the 5′ end-specific cap structure of eukaryotic poly-A mRNAs have been devised [9-14]. All these approaches however require many enzymatic and purification steps and as a consequence, relatively large quantities of starting poly-A mRNAs are needed which, once again, are difficult to obtain from environmental samples where eukaryotic mRNAs are diluted among predominant bacterial RNAs. cDNA size fractionation by agarose gel electrophoresis is an alternative strategy which requires few enzymatic steps and allows the preparation of different sized cDNA libraries from a single RNA sample. Libraries enriched in long cDNAs were for example constructed by agarose gel mediated size fractionation of cDNA synthesized from mouse embryo and human brain [15,16]. However, despite the application of all these strategies, all of them also require microgram amounts of poly-A mRNAs and numerous clones in these cDNA libraries still represent truncated transcripts.

Reverse transcriptase (RT) template switching is another approach for generation of cDNAs resulting from the reverse transcription of entire RNAs [17]. This technique, implemented in the commercial SMART (Clontech) or Mint (Evrogen, Moscow, Russia) kits, makes use of two activities of MMLV RT. The first one is to add a few deoxycytidines (dC) at the end of single strand (ss) cDNAs. The second one is to switch template and to reverse transcribe an oligonucleotide whose 3′ deoxyguanosine-rich sequence anneals to the dC stretch artificially added at the end of the ss cDNA. As a consequence all cDNAs are bordered at their 3′ end by the same oligonucleotide sequence which can be used for their amplification by PCR in combination with a primer sequence added to the poly-dT primer used to initiate reverse transcription the mRNA.

In the present investigation, we demonstrate an efficient method of construction of sized eukaryotic cDNA libraries from environmental samples such as soil. This method is adapted from Wellenreuther *et al*. [18] who combined template switching in combination with agarose gel mediated size fractionation prior to full cDNA PCR amplification to isolate long full-length genes using microgram amounts of purified human poly-A mRNA. In this study, we modified this method to accommodate small limiting amounts of total environmental RNA.

## Results and discussion

### Eukaryotic cDNA synthesis from total soil RNA

We extracted total RNA from three soil samples coming from contrasted geographic localities (Additional file 1). The presence of sharp bands in the Bioanalyser electrophoregram, corresponding to small and large subunits rRNA and of a wide mRNA smear (approx. from 0.2 kb to 5 kb) suggested that these RNA samples were not degraded (data not shown). Extraction yields ranged from 330 to 980 ng.g^-1^ of soil and at least 3 μg of total soil RNA were obtained from each sample.

According to Urich *et al*. [19], soil RNA can be constituted of up to 90% of non-coding sequences (essentially rRNA) and approximately only 7% of the coding sequences can originate from eukaryotes. Although these figures certainly differ from one soil to another, bacterial biomass seems to always dominate in soil [20]. As a consequence, purification of μg amounts of poly-A mRNAs, as recommended in most cDNA library construction protocols, can hardly be met. We therefore developed a protocol which makes use of (i) few μg of total RNA as starting material and (ii) includes long range PCR amplification for the synthesis of long cDNAs. This protocol, implemented in the Mint-2 kit (Evrogen) allowed us to obtain ds cDNAs of eukaryotic origin from as low as 3 μg of total soil RNA. Such a quantity may contain only a few ng of poly-A mRNA. Success of cDNA synthesis was demonstrated by PCR amplification of an EF1α gene fragment (data not shown).

### Size fractionation and PCR amplification of eukaryotic cDNAs

**Figure 1:**
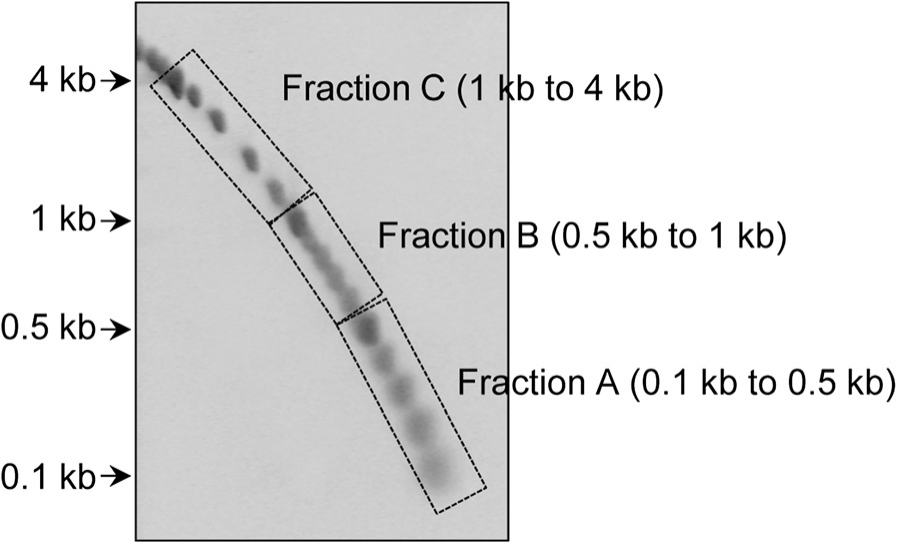
**Bi**-**dimentional cDNA size fractionation.** A DNA size standard was run along with cDNA on two identical but separate agarose gels to visualize the fractionation. The cDNAs unstained gel was superimposed over the ethidium bromide-stained DNA marker gel and three gel slices containing different cDNA size fractions were cut out.

**Figure 2:**
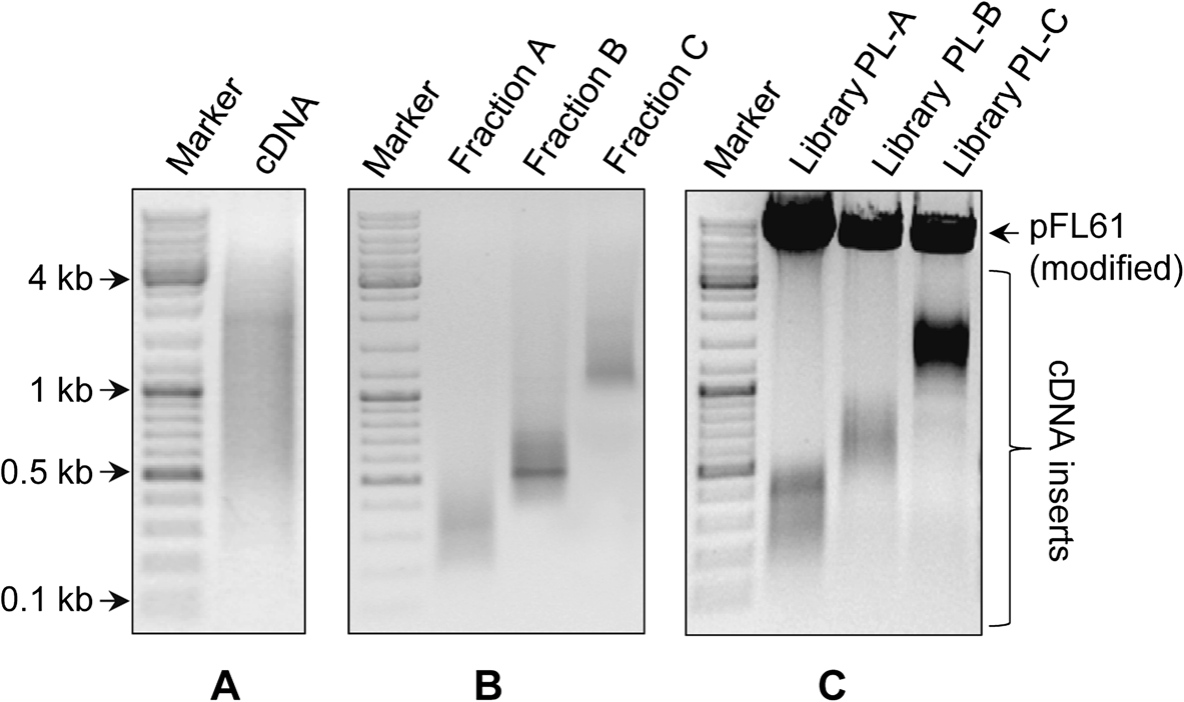
**cDNA size fractions and libraries preparation from soil PL.** Agarose gel separation of **(A)** amplified cDNA without size fractionation, **(B)** the three different cDNA fractions A (0.1–0.5 kb); B (0.5–1 kb) and C (1–4 kb) and **(C)***Sfi*I digested libraries PL-A, PL-B and PL-C. For each sized library, the size range of the cDNA inserts is similar before **(B)** and after **(C)** cloning.

**Figure 3:**
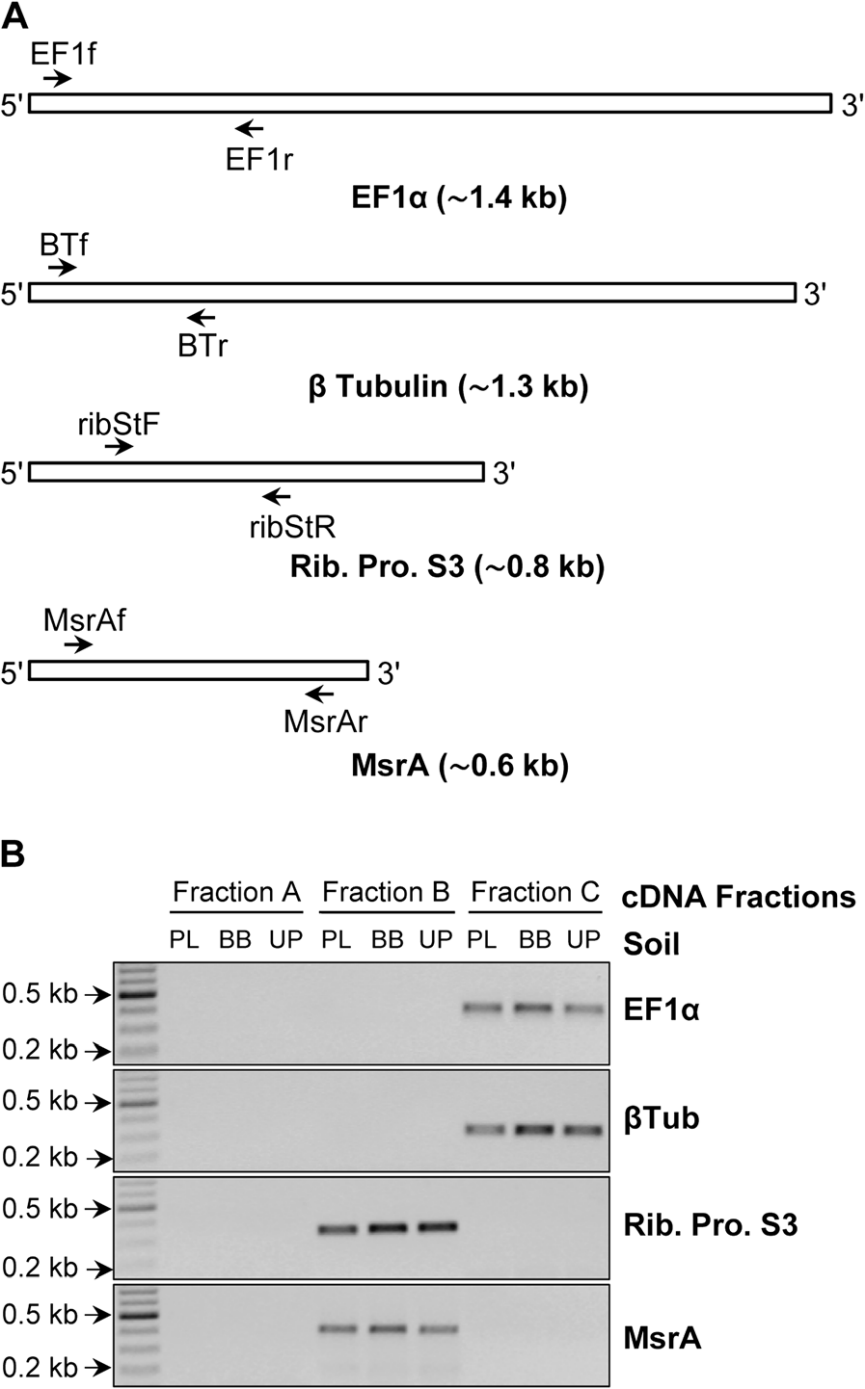
**PCR validations of each cDNA size fraction obtained from soil samples PL**, **BB and UP.** All three cDNA size fractions from each soil were tested for cross-contaminations by PCR amplification of conserved genes of different length. **(A)** Approximate size of coding sequences of each of the four tested genes and positions of the PCR primers. **(B)** Gel electrophoresis of the PCR products showing perfect correlation between sized cDNA fraction and detection of a PCR product from genes whose expected size is included in the corresponding fraction size range. In fraction C, only, elongation factor 1-alpha and beta-tubulin genes were amplified while in fraction B, only ribosomal protein S3 and peptide methionine sulphoxide reductase genes were detected whereas none of theses genes was amplified from fraction A.

Getting a high number of clones having long inserts (e.g. above 1 kb) is one of the main challenges in cDNA library construction. As suggested in [18], we performed size fractionation of cDNA prior to their amplification to facilitate both the synthesis of long cDNA and minimize the preferential amplification of short ones from complex mixtures of long and short cDNAs. In the current protocol, ds cDNAs were first amplified through three PCR cycles, to slightly increase cDNA quantities without affecting significantly their average length. Pre-amplified cDNAs were then separated by bi-dimensional agarose gel electrophoresis (Figure 1) to maximize complete separation of cDNAs and minimize cross-contamination of size fractions. The cDNA containing gel was left unstained to prevent the adverse effect of UV irradiation but also because of the low quantity of loaded cDNAs which prevented their visualization. Three different size fractions (A, 0.1–0.5 kb; B, 0.5–1 kb and C, 1–4 kb) were recovered and amplified by PCR using between 22 and 30 cycles. While amplified cDNAs without size fractionation gave a broad smear throughout the gel lane (Figure 2A), three distinct and discrete DNA smears (broad bands) corresponding to the different cDNA fractions were clearly visible (Figure 2B and Additional file 2A & B). Fractions were tested for the absence of cross contaminations by PCR amplification of four eukaryotic genes of different length classes. For the 1–4 kb class, the conserved genes encode β-tubulin (β-Tub) and EF1-α whose coding sequences are of about 1300 and 1400 bp respectively, while for the 0.5–1 kb class, they encode 40S ribosomal protein S3 (RibS3) and peptide methionine sulphoxide reductase (MsrA) whose coding sequences are of about 800 and 600 bp respectively. As expected, positive amplifications of β-Tub and EF1-α fragments were only obtained for fractions C (1–4 kb), while RibS3 and MsrA fragments were only detected for the intermediate B fractions (0.5–1 kb) and no amplification was visible for the lowest A fractions (0.1–0.5 kb) (Figure 3). These results demonstrate that the method can not only be used for obtaining long cDNAs but also in getting high quality eukaryotic cDNA size fractions from very low amounts of starting total RNAs.

### Construction of sized cDNA libraries

The three cDNA fractions from soil sample PL and the largest fraction C from sample BB were used for construction of cDNA libraries by directional cloning in the pFL61 yeast expression vector. All four libraries contained at least 10^6^ independent clones (range from 1.1 10^6^ for library PL-B to 2.6 10^6^ for library BB-C), but many more could have been obtained. After *Sfi*I digestion of the library plasmid pools and their separation by agarose gel electrophoresis, the released cDNA insert pools of each library were detected as a smear whose size range corresponded to the original cDNA fraction size (Figure 2C and Additional file 2C). The cDNA inserts of 42 random colonies from each of the three libraries from PL soil and 30 from library BB-C were PCR amplified. Absence of inserts was found for less than 1% of the plasmids and all of the PCR products fell within their expected size range (Additional file 3). For the 3 PL libraries, ten inserts per library were sequenced from their 5′-ends. Globally, 60% of the sequences returned a positive result upon BlastX searches against the Eukaryotic GenBank protein database (80%, 70% and 30% for inserts from libraries PL-C, PL-B and PL-A respectively; Additional file 4).

## Conclusions

We established a robust protocol to generate high quality eukaryotic sized cDNA libraries from total RNA extracted from environmental samples. We demonstrated that the original protocol developed by Wellenreuther *et al*. [18] using micrograms amounts of purified poly-A mRNA extracted from human tissues can be considerably scaled down and implemented on total RNA from environments dominated by bacterial RNA. This protocol fulfils several requirements. Firstly yields of RNA extraction directly from environments are usually low (sometimes less than 100 ng.g^-1^ of soil) and therefore isolation of μg amounts of poly-A mRNA appears, in most cases, almost unfeasible. Secondly, two dimensional electrophoretic separation of cDNAs leads to isolation of sized cDNA pools almost free from contaminations by either longer or shorter cDNAs. Most importantly these pools of amplified cDNAs allow production of large cDNA libraries necessary to capture the gene diversity which characterizes microbial communities. Thirdly, the production of sized cDNA libraries is of direct relevance in the context of functional metatranscriptomics as it should reduce the screening effort for gene categories of defined length which, as a result of size selection, will not be diluted among shorter or longer transcripts. As an example, most glycoside hydrolases (e.g. cellulases, hemicellulases), implicated in organic matter degradation, are encoded by transcripts 1–3 kb in length and screening for these genes can be limited to the corresponding sized cDNA libraries. In conclusion, we believe that the protocol presented in this paper should facilitate and promote studies of environmental eukaryotic communities, which in a context of environmental biotechnology represent a promising and almost untouched source of genes of interest.

## Methods

### RNA extraction from soil

Three soils, two from France (PL and BB) and one from India (UP) were used (Additional file 1). Total RNAs were isolated from soil samples according to Damon *et al*. [21] for the BB sample or by using the RNA PowerSoil® Total RNA Isolation Kit (Mo Bio laboratories, Carlsbad, CA) for the PL and UP samples. All soil RNA samples were treated with RNase-free DNase I. After a final precipitation step, all the RNAs were dissolved in nuclease-free water. RNA integrity was checked by Bioanalyzer 2100 (Agilent Technologies, USA) electrophoresis and RNA quantity and purity were determined by spectrophotometry (SAFAS UVmc2, SAFAS Monaco).

### Synthesis, size fractionation and amplification of cDNAs

cDNAs were synthesized by using the Mint-2 cDNA synthesis kit (Evrogen, Moscow, Russia) according to the manufacturer’s instructions. Briefly, three μg of total soil RNAs were mixed with 10 μM of two oligonucleotide adapters. The 3′end CDS-4M adapter contains (i) an oligo (dT) sequence that anneals to the poly (A) stretch of eukaryotic mRNAs, (ii) a *Sfi*IB restriction site and (iii) sequence of primer M1. The 5′-end PlugOligo-3M adapter contains (i) an oligo (dG) sequence which anneals to the complementary oligo (dC) stretch added to the 3′-end of the first-strand cDNA by Mint MMLV RT (ii) a *Sfi*IA restriction site and (iii) sequence of primer M1. The mixture was incubated at 70°C for 2 minutes. First-strand cDNAs were synthesized at 42°C by Mint RT in presence of dNTPs, DTT, first-strand buffer and IP-solution in 15 μL of total reaction volume. Second-strand cDNA synthesis was carried out by the thermostable Encyclo DNA polymerase (Evrogen) using the M1 primer which recognizes both the PlugOligo-3M and CDS-4M adapter sequences. Four μL from first-strand cDNA reaction, i.e. the equivalent of 800 ng of total soil RNA, were used in second-strand cDNA synthesis. Second strand synthesis was followed by a PCR amplification limited to 3 cycles at 95°C for 15 sec, 66°C for 20 sec and 72°C for 3 min. Resulting double-stranded cDNAs (ds cDNAs) were purified by phenol-chloroform extraction and precipitated.

Size fractionation of cDNAs was performed as described by Wellenreuther *et al*. [18]. ds cDNAs and a DNA size standard were size fractionated in two separate but identical 0.7% agarose gels at identical running conditions. After electrophoresis, the gel lanes containing ds cDNAs and DNA size standard were cut out, rotated at 90° and placed into two separate but identical gel trays. Identical volumes of 1.4% low melting point agarose (Bioprobe Systems, Montreuil, France) were cast in both the trays and the gels were subjected to electrophoresis at 2.6 V.cm^-1^ for 10 h. The unstained gel containing cDNAs was then superimposed over the ethidium bromide stained gel containing the size standard which was visualized using a Dark Reader transilluminator (Clare Chemical Research, Inc., USA). Three gel slices corresponding to the cDNA size fractions A, 0.1–0.5 kb; B, 0.5–1 kb and C, 1–4 kb, were cut out from the unstained gel. cDNAs were extracted from each gel slice by using QIAEX II Gel Extraction Kit (Qiagen, Netherlands), precipitated and amplified by PCR using primer M1 as described above but using higher number of cycles. Depending on cDNA size fraction and RNA sample, between 22 to 30 cycles were performed for optimal amplification.

### Validation of cDNA size fractions

Validation of the different sized cDNA fractions was performed by running each of them in standard 1% agarose gels and by PCR amplification, on each of them, of different gene families representative of the different size groups. For fraction C, the selected families encoded β-Tubulin and Elongation factor 1-alpha (EF1α). They were amplified using respectively primer pairs BTf (GGTAACCAAATCGGTGCTGCTTTC)/BTr (ACCCTCAGTGTAGTGACCCTTGGC) [22] and EF1f (GTCGTYGTYATYGGHCAYGT)/EF1r (TGYTCNCGRGTYTGNCCRTCYTT) [23]. For fraction B, the selected families encoded 40S Ribosomal protein S3 amplified using primers ribStF (CHSKHACYGABRTCATCATCCG) and ribStR, (AADCCRTCRGTGAACTTCATG) (this study) and Peptide methionine sulphoxide reductase amplified using MsrAf (CGCCGCCGGCTGYTTYTGGGG) and MsrAr (ATRGTRGTYNWCATGGACCTGTTCTTGGGGC) [24]. For the smallest fraction A, no conserved gene family in this size range could be identified to design PCR primers that would work on environmental cDNA samples. Ten ng of each cDNA fraction were used as template in 35 cycles PCR reactions.

### cDNA cloning

cDNA fractions (500 ng) were digested by *Sfi*I which recognizes *Sfi*IA and *Sfi*IB sites located in the sequences of PlugOligo-3M and CDS-4M, respectively. Following phenol-chloroform extraction and precipitation, cDNAs were ligated downstream of the *S. cerevisiae* PGK1 promoter in a modified pFL61 yeast expression vector containing *Sfi*IA and *Sfi*IB sites [5,25]. Recombinant plasmids were introduced into electro-competent *E. coli* cells (MegaX DH10B™ T1R Electrocomp™ cells, Invitrogen) and at least 10^6^ ampicillin resistant bacterial colonies growing on agar medium were collected and pooled to constitute each of the libraries.

### Sequencing

Plasmids were isolated from ten randomly selected bacterial colonies from each of the three libraries constructed from PL sample. cDNA inserts were sequenced from their 5′ end and deduced amino acid sequences were used in similarity search (BLASTX) against the GenBank nr eukaryotic protein database (as in December 2013). Resulting cDNA sequences appear in the EMBL database under accession Nos. HG964498 to HG964527.

## Competing interests

The authors declare that they have no competing interests.

## Authors’ contributions

RKY, RM and LF designed and coordinated the study. RKY, FB, AZ and MSR carried out the experiments. RKY, RM and LF wrote the manuscript, and PL and MSR reviewed the manuscript. All authors read and approved the final manuscript.

## Supplementary Material

Additional file 1 Main characteristics of the soil sampling sites. Click here for file

Additional file 2 **cDNA size fractionation from different soils.***Description of data*: Agarose gel showing amplified eukaryotic cDNA fractions **A**, **B** and **C** after size fractionation from **(A)** Indian soil UP and **(B)** French forest soil BB sample. Fraction C of soil BB was cloned in modified pFL61 vector. **(C)** The corresponding library (BB-C) was digested with *Sfi*I enzyme realeasing cDNA inserts of sizes ranging, as expected, between 1 and 4 kb. Click here for file

Additional file 3 **Validation of insert size by colony PCR amplification.***Description of data*: cDNA inserts from 42 random colonies from each of the three libraries PL-A, PL-B and PL-C and 30 random colonies from library BB-C were amplified by colony PCR. As expected, the sizes of amplified DNA inserts of each library were confined between their expected size cut offs. Panels **A**, **B** and **C** are the gel images of separated PCR products from libraries PL-A, PL-B and PL-C respectively. Panel **D** is the gel image after migration of PCR products from library BB-C. Click here for file

Additional file 4 **Protein similarity search of sequenced cDNA inserts using blastx against GenBank eukaryotic protein sequences.** An e-value threshold of 10^-5^ was retained for annotation. aa, amino acids. Click here for file
