# Supplementary material for: Construction of sized eukaryotic cDNA libraries using low input of total environmental metatranscriptomic RNA
Source: BMC Biotechnol. 2014 Sep 3;14:80. doi: 10.1186/1472-6750-14-80 (PMC4170940; doi:10.1186/1472-6750-14-80)
Supplement: Additional file 1 — Main characteristics of the soil sampling sites. [file 1472-6750-14-80-S1.pdf]

**Additional file 1.** Main characteristics of the soil sampling sites

| Soil Samples | PL | BB | UP |
|---|---|---|---|
| **Coordinates** | 49°1'45''N, 2°10'32''E | 47°18'10''N, 4°4'44''E | 24° 22' 48"N, 73° 45' 24"E |
| **Climate type** | semi-oceanic temperate | Continental temperate | Tropical monsoon continental |
| **Vegetation** | Poplar plantation (*Populus. sp.*) | Beech forest (*Fagus sylvatica*) | *Acacia Senegal*, *Delbergia sissoo*, *Azadirachta indica*, *Bougainvillea glabra, Thevetia peruviana* |
| **Mean annual Temperature (°C)** | 10.9 | 6 | 22.7 |
| **Mean annual rainfall (mm)** | 630 | 1400 | 637 |
| **Soil type** | Sandy luvisol soil | Alocrisol | Sandy Ustochrepts soil |
| **Soil pH** | 7.1 | 4.5 | 7.5 |
| **Organic carbon (%)** | 1.6 | 7.73 | 2.27 |
| **Sampling date** | July 4, 2012 | July 10, 2007 | Oct. 19, 2012 |
| **Soil temperature (°C)*** | 24 | 14.4 | 35 |
| **Soil water content (%)*** | 5.8 | 22.5 | 1.5 |
| **Parental material** | Limestone | Granite | Sphalerite (Zinc sulfide) |

* On the sampling day
