# Supplementary figures and images for: Construction of sized eukaryotic cDNA libraries using low input of total environmental metatranscriptomic RNA
Source: BMC Biotechnol. 2014 Sep 3;14:80. doi: 10.1186/1472-6750-14-80 (PMC4170940; doi:10.1186/1472-6750-14-80)

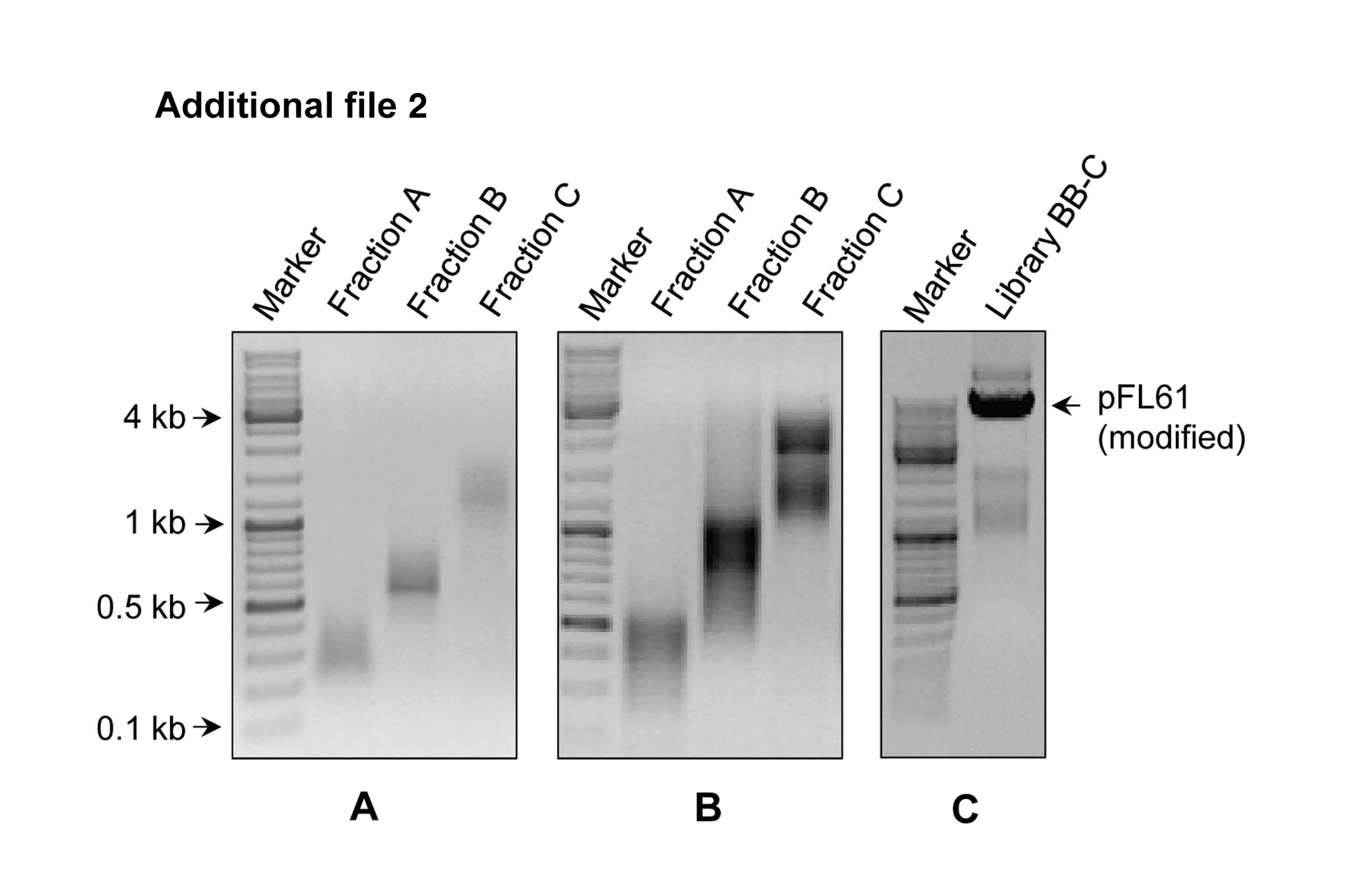

Supplement: Additional file 2 — cDNA size fractionation from different soils.Description of data: Agarose gel showing amplified eukaryotic cDNA fractions A, B and C after size fractionation from (A) Indian soil UP and (B) French forest soil BB sample. Fraction C of soil BB was cloned in modified pFL61 vector. (C) The corresponding library (BB-C) was digested with SfiI enzyme realeasing cDNA inserts of sizes ranging, as expected, between 1 and 4 kb. [file 1472-6750-14-80-S2.tif]

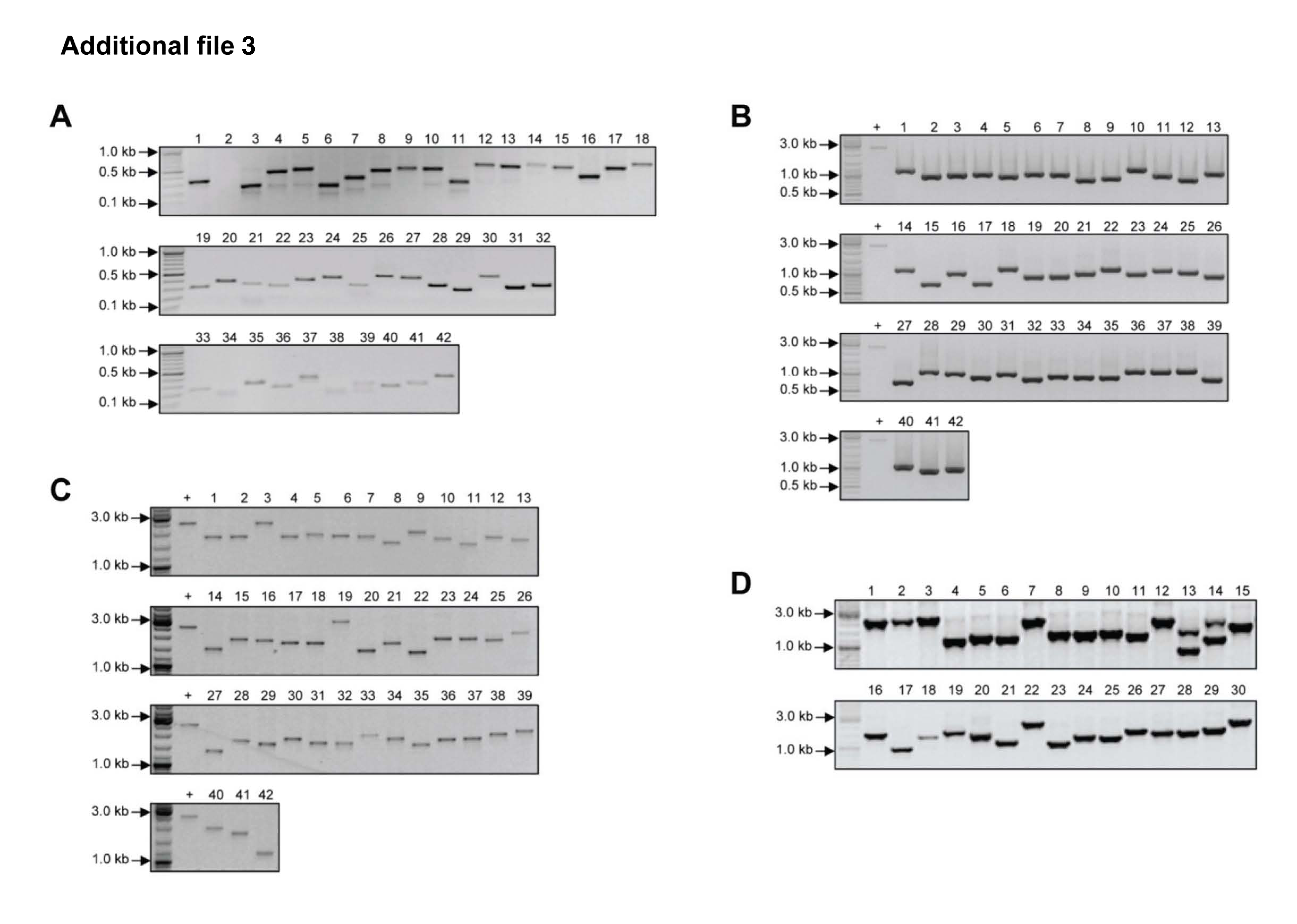

Supplement: Additional file 3 — Validation of insert size by colony PCR amplification.Description of data: cDNA inserts from 42 random colonies from each of the three libraries PL-A, PL-B and PL-C and 30 random colonies from library BB-C were amplified by colony PCR. As expected, the sizes of amplified DNA inserts of each library were confined between their expected size cut offs. Panels A, B and C are the gel images of separated PCR products from libraries PL-A, PL-B and PL-C respectively. Panel D is the gel image after migration of PCR products from library BB-C. [file 1472-6750-14-80-S3.tif]
