# Supplementary material for: Construction of sized eukaryotic cDNA libraries using low input of total environmental metatranscriptomic RNA
Source: BMC Biotechnol. 2014 Sep 3;14:80. doi: 10.1186/1472-6750-14-80 (PMC4170940; doi:10.1186/1472-6750-14-80)
Supplement: Additional file 4 — Protein similarity search of sequenced cDNA inserts using blastx against GenBank eukaryotic protein sequences. An e-value threshold of 10-5 was retained for annotation. aa, amino acids. [file 1472-6750-14-80-S4.pdf]

Additional file 4. Protein similarity search of sequenced cDNA inserts using blastx against GenBank eukaryotic protein sequences. An e-value threshold of 10^{-5} was retained for annotation. aa, amino acids.

Clone no.	Accession no.	Annotation	e-value	Alignment length (aa)	% Identity (aa)	% Positive (aa)
**Library PL-A**						
A-1	HG964498	No significant similarity found	-	-	-	-
A-2	HG964499	No significant similarity found	-	-	-	-
A-3	HG964500	Hypothetical protein	8.00E-27	77	77	83
A-4	HG964501	No significant similarity found	-	-	-	-
A-5	HG964502	No significant similarity found	-	-	-	-
A-6	HG964503	Thioredoxin	1.00E-05	25	88	96
A-7	HG964504	No significant similarity found	-	-	-	-
A-8	HG964505	No significant similarity found	-	-	-	-
A-9	HG964506	Hypothetical protein	3.00E-10	89	53	60
A-10	HG964507	No significant similarity found	-	-	-	-
**Library PL-B**						
B-1	HG964508	ABC transporter	2.00E-29	123	49	69
B-2	HG964509	Predicted protein	2.00E-29	101	69	78
B-3	HG964510	60S ribosomal protein L32	5.00E-76	131	92	95
B-4	HG964511	No significant similarity found	-	-	-	-
B-5	HG964512	Hypothetical protein	6.00E-22	66	76	77
B-6	HG964513	Hypothetical protein	7.00E-14	60	52	66
B-7	HG964514	Ceramide kinase-like	1.00E-07	59	44	71
B-8	HG964515	No significant similarity found	-	-	-	-
B-9	HG964516	No significant similarity found	-	-	-	-
B-10	HG964517	Predicted protein	3.00E-21	129	39	55
**Library PL-C**						
C-1	HG964518	Hypothetical protein	7.00E-64	247	44	66
C-2	HG964519	Hypothetical protein	5.00E-70	263	44	66
C-3	HG964520	Hypothetical protein	1.00E-33	197	37	57
C-4	HG964521	Pro-apoptotic serine protease nma111	4.00E-160	270	90	96
C-5	HG964522	No significant similarity found	-	-	-	-
C-6	HG964523	Hypothetical protein	2.00E-98	257	58	71
C-7	HG964524	Hypothetical protein	4.00E-29	122	49	67
C-8	HG964525	Na+/H+ antiporter	3.00E-20	191	35	56
C-9	HG964526	No significant similarity found	-	-	-	-
C-10	HG964527	Aldehyde dehydrogenase domain containing protein	3.00E-111	222	71	85
